# Obturator Prosthesis Rehabilitation after Maxillectomy: Functional and Aesthetical Analysis in 25 Patients

**DOI:** 10.3390/ijerph182312524

**Published:** 2021-11-28

**Authors:** Massimo Corsalini, Giuseppe Barile, Santo Catapano, Annamaria Ciocia, Assunta Casorelli, Rosaria Siciliani, Daniela Di Venere, Saverio Capodiferro

**Affiliations:** 1 Department of Interdisciplinary Medicine, University of Bari “Aldo Moro”, 70124 Bari, Italy; g.barile93@hotmail.it (G.B.); anna.ciocia1@gmail.com (A.C.); a.casorelli1@studenti.uniba.it (A.C.); aricleo@libero.it (R.S.); daniela.divenere@uniba.it (D.D.V.); 2 Dental Clinic, Dental School, University of Ferrara, 44121 Ferrara, Italy; santo.catapano@unife.it

**Keywords:** maxillary obturator prosthesis, maxillary neoplasms, post-surgical complications, quality of life

## Abstract

The number of patients undergoing a surgical resection of the maxilla for oncological reasons is constantly increasing, the most common complication of which remains the communication between oral and nasal cavities. On the basis of data arising from the literature regarding the treatment options of maxillary oncological post-surgical defects, obturator prosthesis remains the most used worldwide. We studied 25 patients (with at least 1-year follow up) rehabilitated by obturator prosthesis after maxillary resection leading to oro-nasal communication, providing data on the objective/subjective evaluation of such rehabilitation and mastication performance measured by a two-color chewing gum test. The type of defect was classified according to the classification system proposed by Aramany. Among the patients in our study, 72% rated a higher score for either stability and retention than for aesthetic appearance, as confirmed by the Kapur score rated by clinicians. The two-color chewing gum test shows similar results as only one patient had insufficient chewing function. Interestingly, we found no correlation between the masticatory function and residual denture, confirming that the maxillary obturator remains a predictable solution in such patients regardless of the anatomical alterations following surgery.

## 1. Introduction

Maxillary defects are generally classified as congenital (e.g., palate clefts) and acquired, the latter usually following the surgical resection of both maxillary benign or malignant (epithelial and salivary) neoplasms, infectious diseases or bone osteonecrosis [1,2,3,4,5,6,7,8,9]. As for malignancies, head and neck cancers (HNCs) represent 5% of all malignancies in the Italian population—90% of which are oral squamous cells carcinomas (OSCCs) involving the hard and soft palates in less than 13% of cases. Additionally, salivary gland neoplasms, which represent approximately 3% of all head and neck neoplasms, may involve the palate when occurring in the minor salivary glands [9,10,11,12]. Partial or total maxillectomy is usually indicative of such malignancies, frequently resulting in a communication between the nasal and oral cavities when direct closure and/or reconstruction is not indicative or impossible to perform. In such cases, several functional complications may become manifest, such as difficulties in chewing, biting and speaking following hypernasal speech, fluid leaking into the nasal cavity as well as impaired masticator function.

Generally, microvascular free flaps are the most used surgical procedures for defect closure, especially in small ones, although they are generally associated with increased hospitalization and high morbidity in the flap donor area [13].

With the purpose of providing guidelines for oral/maxilla-facial decisive tumor eradication following prosthetic rehabilitation, in 1978, Aramany classified maxillary defects into six different classes as follows: in class I defects, dentition and alveolar bone have been removed along the midline; in class II, the premaxilla on the defect side has been preserved; in class III, the defect is located in the middle of the palate and dentition is preserved (which surely represents the most favorable condition as teeth provide retention to the obturator); in class IV, the defect includes the premaxilla on the side opposite the surgery; in class V, the anterior teeth are preserved while the posterior teeth, hard palate and a variable portion of the soft palate have been resected; in class VI defects, the premaxilla is involved, mostly related to accidental trauma and then surgical resection [14,15].

Nevertheless, as stated by Alì et al. in 2018, regardless of the prosthetic planning for different classes, there is no direct association between the defect type and patient quality of life (QoL) [16].

All the aforementioned clinical situations highlight the importance of the prosthodontic for restoring oral function and aesthetics in a multidisciplinary approach to an HNC patient with the aim of psychological rehabilitation and restoring the patient’s social life [13].

The most common approach to the rehabilitation of patients with maxillary defects remains the use of a removable prosthetic as a defect obturator, precisely defined by The Glossary of Prosthodontics Terms as “a prosthesis used to close a congenital or an acquired tissue opening, primarily of the hard palate and/or contiguous alveolar structures” [17,18]. Among all the generally accepted advantages resulting from their use, such as reduction in hospitalization time and cost, the possibility of avoiding or deleting a second surgical procedure for defect closure and the immediate reestablishment of facial morphology and oral functions is of paramount,—not least for the possibility of a simplified clinical examination of the surgical defect to detect clinical signs of malignancy recurrence at an early stage [19,20,21].

Summing up such considerations, also consistently with the relevant international literature, it can be assumed that obturator prosthesis still represents the gold standard for maxillary defects’ restoration [22].

In the current study, the authors described the protocol used at the Dental School of the University Hospital of Bari “Aldo Moro” for functional and/or aesthetic restoration by obturator prosthesis in oncologic patients following maxillectomy.

## 2. Materials and Methods

Patients treated for malignancy of the palate by surgical resection without flap reconstruction at the University Hospital of the University of Bari “Aldo Moro”, during the period from 2015 to 2020, were selected and enrolled in the current study if they matching the following criteria: surgery performed by the same surgeon; different typologies of surgery and subsequent different sequela (palatal and/or maxillary defects and residual dentition variously represented). Adjunctive exclusion criteria were pre-existing congenital maxillary defects; patients refusing treatment or missing the follow up (every 24 months).

**Figure 1:**
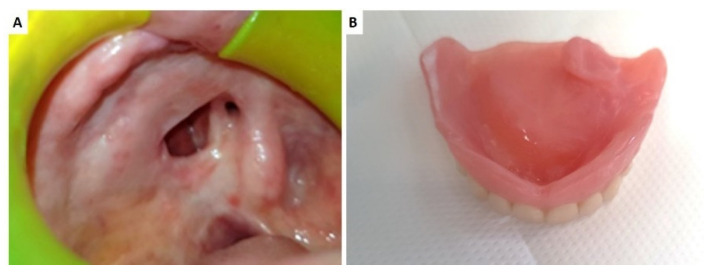
Palatal perforation after maxillectomy for minor salivary gland basal cell carcinoma (**A**); the final prosthesis providing the obturation of the oro-antral communication (**B**).

As for the patient classification, the guidelines proposed by Aramany have been applied and the following timeline respected:

- Time 0: alginate impression to manufacture a pre-surgical obturator in thermoplastic resin;
- Time 1: surgical resection and contextual pre-surgical obturator positioning filled with periodontal dressing (Coepack); in the post-operative course, the obturator was continuously dressed to guide wound healing and exclusively removed for the removal of stiches;
- Time 2: alginate impression to fabricate a new “ad interim” obturator, with a false palate and false ridge and no retention on teeth but filling the defect;
- Time 3: at the time of complete wound healing and defect dimension stability, the silicon was impressed to fabricate the definitive obturator (Figure 1).

After the last general check (including prosthesis fitting, retention, stability, centric and eccentric movement analysis and aesthetic acceptance), prosthesis was delivered to the patients. At the first follow up (one month later), the patients answered the questionnaire (3 questions) for a subjective evaluation as follows: question 1—anamnestic data and opinion about the prosthesis to rate the aesthetic, retention and stability aspects with a score from 1 to 10; question 2—functional evaluation (chewing of different foods and speaking abilities, assigning a score from 1 to 10; question 3—evaluation of QoL (social life, relationships, humor and self-esteem) assigning a score from 1 to 4. An objective evaluation of obturator retention and stability was assessed by the scoring system described by Kapur (score from 0 to 3 for retention and from 0 to 2 for stability). Masticatory performance (MP) was evaluated by the two-color chewing gum test (5 strips, 30 mm length azure color and pink color chewing gums, consisting of a double bi-chromatic layer), which analyzed the degree of chromatic mixing of the two different colors of the gum after a predefined sequence of chewing cycles, intermixed with a time interval of 1 min to decrease muscle fatigue as follows: first test—5 chewing cycles; second test—10 chewing cycles; third test—20 chewing cycles; fourth test—30 chewing cycles; and fifth test—50 chewing cycles.

**Figure 2:**
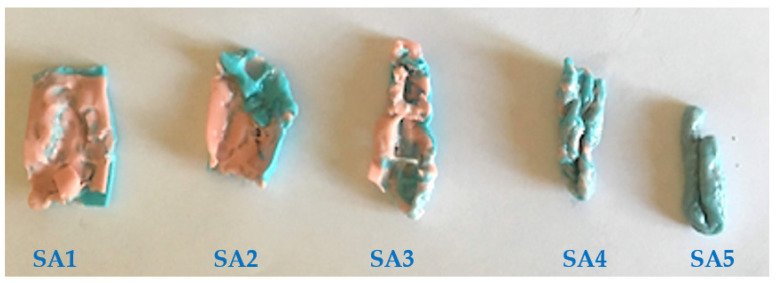
Five different SA (from SA1 to SA5) grades showing the variable mixing of the differently colored chewing gums obtained during masticatory function tests.

The test results were independently evaluated by two different operators to categorize subjective assessment (SA) patients as such (Figure 2):

- SA1: double-layer colors were not mixed; only teeth cusps’ impressions were observable;
- SA2: Most of the double-layer colors was not mixed;
- SA3: A large part of the double-layer colors was mixed;
- SA4: The mixing of colors was observable in the whole sample but not uniform;
- SA5: The color distribution was perfectly uniform.

## 3. Results

Among the 29 patients included in the study, 4 of them did not complete the follow-up visits and were thus discarded. Fourteen males and eleven females were the sex distribution and the mean age was 63.5 years in an overall range between 48 and 79 years. Maxillary defects were distributed as follows: 11 patients in Aramany class I, 4 with class III, 6 with class IV and 4 with class VI. Ten were dentate and fifteen edentulous.

The results of the questionnaire were as follows: 16 patients (64%) presented good oral hygiene status and 9 (36%) bad; 14 patients (56%) had been smokers until the diagnosis of cancer and 4 (16%) were still smokers (an average of 4–5 cigarettes/day); 7 patients (28%) denied current or previous smoking habit; 15 patients (60%) have never used alcohol, 7 (28%) consumed alcohol occasionally and 3 (12%) had been consumers of significant amounts of alcohol until surgery. Subjective evaluation regarding the aesthetic, retention and stability aspects yielded results as follows: a low score (1–5 points) was rated by 7 patients (28%) and a high score (6–10 points) by 18 (72%). The mean retention score was 2.4/3 points and the mean stability score was 1.6/2 points. As for the subjective evaluation of the stomatognathic function, five patients rated 0, nine rated 1, four rated 2, four rate 3 and three rate 4 points (mean scoring was 1.64/4). As for the results of the objective evaluation by the two-color chewing gum test (Figure 2), 1 patient showed an SA1 grade (4%), 6 patients showed an SA2 grade (24%), 8 patients showed an SA3 grade (32%) and 10 patients showed an SA4 grade (40%).

With regard to the QoL evaluation, one patient reported very low QoL (4%), three reported bad QoL (12%), nine reported good QoL (36%), eight reported satisfactory QoL (32%) and the remaining four reported excellent QoL (16%). A statistical analysis was conducted: the Shapiro–Wilk normality test showed that the data distribution was not normal, and for such reason, the Spearman test was used to correlate the (*p* < 0.05) SA scoring with the Aramany classes and dentition after surgery. A significant correlation was found between the SA score and type of dentition (*p* = 0.03393) while no statistical correlation was detected between the type of defects and SA score (*p* = 0.42936). All data are summarized in Table 1.

## 4. Discussion

Among oral cavity neoplasms, 1–5% occur in the hard palate and upper gingiva, the most frequent being the OSCC—especially in adults (usually in the sixth to seventh decades of life) [9,23]. Surgical resection with or without adjuvant chemotherapy and/or radiotherapy is the most common approach for palatal malignancies, with the following microvascular flap reconstruction in a limited number of cases, thus possibly leading to hypernasal speech, liquid leaking into the nasal cavities, drooling, impaired mastication and aesthetic complications for reduced or altered support to the oro-facial soft tissues in all the un-reconstructed post-surgically remaining cases because of a variably sized perforation of the palate/maxilla [22]. No less importantly, such patients suffer the progressive deterioration of their relationship life due to low self-esteem [19].

Therefore, for all the aforementioned reasons, the most common approach to the functional, aesthetical and psychological rehabilitation of a patient with palatal/maxillary post-surgical defects remains the use of a removable prosthetic as obturator [17,24,25]. The history of maxillary obturator prostheses is well documented, especially in congenital rather than acquired defects. Ambroise was the first to use an artificial device to close a palatal defect as early as the 1500s; Claude Martin described the use of a surgical obturator prosthesis in 1875, while Fry described the use of impressions before surgery in 1927 [26]. The most used construction protocol still used today was first described by Keyf and consists of three phases as follows: surgical, interim and definitive [27]. The obturator in the surgical phase provides a matrix for the placement of the surgical packing to reduce wound contamination and promote the second-intention healing as direct closure is relatively difficult to perform in a relatively high number of cases. The interim obturator prosthesis is usually placed one week later and is easily modifiable by lining material to be better adaptable to surgical wound changes during healing [28]; this is a critical phase as, in absence of prosthetic support, tissue contracture may rapidly promote the collapse of defect borders with hardly correctable unaesthetic and contorted facial contours [29]. Once the maxillary defect remains dimensionally stable, usually after a generally reported median period of 3–4 months after surgery, a definitive maxillary obturator prosthesis can be manufactured. Usually, such prostheses are conventionally made of chromium–cobalt frameworks with poly-methyl-methacrylate for dentate patients or only poly-methyl-methacrylate for edentulous; also, the bulb is preferably lightweight to provide better patient comfort, less pressure to the defect peripheral tissues and more efficiency compared to the solid bulb obturator [30].

In the current study, the mean age of patients was higher than that in other studies, probably due to the high mean population age in the Mediterranean area compared with the rest of the world [31,32,33]. Most cases presented a class I defect, followed by classes IV, VI and III classes, respectively. The same prevalence is generally reported in the literature, with the exclusion of Kumar’s results [16,30,34,35]. The obturator was rated a high score by 72% of patients in the first month with regard to fluid leakage, speech, retention and stability: similar results were observed by Ali and Irish [16,22]. A good to excellent QoL was rated by 84% of respondents according to the generally reported correlation between good obturator retention and enhanced QoL [36,37,38,39]. Moreover, supporting obturator use, the QoL of patients treated with obturator prostheses and that of patients without tumors was similar [23]. Of note are the results of the statistical analysis among our cases. Generally, the two-color chewing gum test is a widely validated method used to objectively measure MP in patients with compromised dentition [40,41,42]. In this regard, the authors compared MP with dentition after surgery and Aramany’s classes of maxillary defects. The literature data agree with regard to the influence of dentition in retention and the stability of the obturator with improved functionality in dentated patients and bad performances in edentulous patients; temporary or definitive alterations of the temporo-mandibular joint movements should also be considered in such patients [31,43,44]. In fact, the number and position of teeth and periodontal status are overall considered the most critical factors to account for the great amount of functional stress that the obturator inflicts on the remaining teeth [27]. A statistically significant correlation between the MP and residual dentition was highlighted by the results of the current study as well as those of several overlapping studies using different methods for MP evaluation [16,45,46]; in this regard, only Kreeft found no correlation between the obturator function and maxillary teeth or dental implants [47,48,49]. Notably, we found no correlation between the MP and type of maxillary defect. Authors claimed that individuals who had a smaller resected palate area had more retentive and stable obturators as well as better QoL than those who had more than one quarter of their palate resected [16,36,45,49]. This result could probably attributed to other variables that influence MP such as the specific planning of obturator construction or continuous follow-up visits that could be useful to improve MP, even when the defect is large, to better preserve the remaining teeth.

## 5. Conclusions

Using dental or zygoma implants as supports for obturator prostheses surely provides a notably improvement in the masticatory function of such patients, but such treatment is not always possible to perform for different reasons (costs, insufficient residual bone, chemo-radio therapies, etc.). The obturator remains the most acceptable prosthetic treatment for oncologic patients with acquired maxillary defects. Masticatory performance depends on residual dentition and good design is mandatory to overcome the type and size of maxillary defects, in order to improve the oncologic patients’ QoL.

## Figures and Tables

**Table 1:** Overall data collected from 25 patients.

Patient	Sex	Age	Aramany	Dentition	Oral Hygiene	Smoking Habits	Alcohol Habits	Subjective Evaluation	Subjective Function Evaluation	SA Score	Quality of Life
1	M	67	I	D	G	Until Cancer	Never	High	3	3	Satisfactory
2	M	54	I	E	G	Yes	Significant	Low	0	2	Good
3	M	62	III	D	B	Until Cancer	Never	High	1	4	Good
4	F	73	I	D	B	Until Cancer	Never	High	2	4	Satisfactory
5	F	48	VI	E	G	Until Cancer	Never	Low	0	2	Good
6	M	65	I	D	B	Yes	Occasionally	High	2	3	Good
7	F	59	IV	E	G	Until Cancer	Occasionally	Low	0	2	Good
8	M	68	I	E	G	Until Cancer	Never	Low	0	1	Very Low
9	F	63	III	D	B	Never	Never	High	3	3	Satisfactory
10	M	73	III	D	B	Until Cancer	Never	High	1	4	Good
11	M	79	IV	E	G	Never	Never	High	3	4	Satisfactory
12	F	60	I	E	G	Until Cancer	Occasionally	High	1	3	Good
13	F	55	IV	E	G	Yes	Significant	Low	1	2	Bad
14	M	64	I	E	G	Until Cancer	Never	High	3	4	Excellent
15	F	72	I	D	B	Never	Never	High	1	3	Good
16	M	57	IV	E	G	Until Cancer	Occasionally	High	2	4	Satisfactory
17	F	66	VI	E	G	Never	Never	Low	1	2	Bad
18	F	61	I	D	B	Never	Occasionally	High	2	3	Satisfactory
19	M	60	VI	E	G	Yes	Never	High	1	4	Satisfactory
20	M	71	III	D	B	Until Cancer	Never	High	1	4	Satisfactory
21	F	68	I	D	B	Until Cancer	Significant	High	4	4	Excellent
22	M	56	IV	E	G	Never	Never	Low	0	2	Bad
23	F	59	IV	E	G	Until Cancer	Occasionally	High	4	4	Excellent
24	M	66	VI	E	G	Until Cancer	Occasionally	High	4	3	Excellent
25	M	62	I	E	G	Never	Never	High	1	3	Good

Legend: D = dentate; E = edentulous; B = bad; G = good.

## Data Availability

Not applicable.

## References

[1] Mirna G., Lokanath G., Aswini K.K., Chittaranjan B. (2010). Prosthetic rehabilitation of a unilateral maxillary defect with an intermediate obturator. Indian J. Dent. Adv..

[2] Capodiferro S., Favia G., Lacaita M., Muzio L.L., Favia G. (2005). Juvenile angiofibroma: Report of a case with primary intra-oral presentation. Oral Oncol. Extra.

[3] Limongelli L., Cascardi E., Capodiferro S., Favia G., Corsalini M., Tempesta A., Maiorano E. (2020). Multifocal amelanotic melanoma of the hard palate: A challenging case. Diagnostics.

[4] Capodiferro S., Calabrese L., Maffini F., Cascardi E., Favia G., Maiorano E. (2019). Dentinogenic ghost cell tumour in a 20-year-old male with previous squamous cell carcinoma of the tongue. J. Biol. Regul. Homeost. Agents.

[5] Limongelli L., Capodiferro S., Tempesta A., Sportelli P., Dell’Olio F., Angelelli G., Maiorano E., Favia G. (2020). Early tongue carcinomas (clinical stage I and II): Echo-guided three-dimensional diode laser mini-invasive surgery with evaluation of histological prognostic parameters. A study of 85 cases with prolonged follow-up. Lasers Med. Sci..

[6] Maiorano E., Favia G., Capodiferro S., Montagna M.T., Lo Muzio L. (2005). Combined mucormycosis and aspergillosis of the oro-sinonasal region in a patient affected by Castleman disease. Virchows Arch..

[7] Tempesta A., Capodiferro S., Mauceri R., Lauritano D., Maiorano E., Favia G., Limongelli L. (2021). Peri-implantitis-like medication-related osteonecrosis of the jaw: Clinical considerations and histological evaluation with confocal laser scanning microscope. Oral Dis..

[8] Di Cosola M., Turco M., Bizzoca G., Tavoulari K., Capodiferro S., Escudero-Castaño N., Muzio L. (2007). Ameloblastoma of the maxillar and mandibular bones: An evidence-based clinical study. Av. Odontoestomatol..

[9] Favia G., Maiorano E., Capodiferro S., Pilolli G.P., Lacaita M.G., Lajolo C., Giuliani M., Martinelli D., Germinario C. (2008). Oral squamous cell carcinoma: A mono-institutional epidemiological study on 462 cases highlighting differences among young and adult patients. Minerva Stomatol..

[10] Van Dijk B.A.C., Gatta G., Capocaccia R., Pierannunzio D., Strojan P., Licitra L. (2012). Rare cancers of the head and neck area in Europe. Eur. J. Cancer.

[11] Ellis G.L., Auclair P.L. (1996). Tumors of the Salivary Glands, Atlas of Tumor Pathology: Third Series, Fascicle 17.

[12] Eneroth C.M. (1969). Incidence and prognosis of salivary-gland tumours at different sites. A study of parotid, submandibular and palatal tumours in 2632 patients. Acta Otolaryngol. Suppl..

[13] de Carvalho-Teles V., Pegoraro-Krook M.I., Lauris J.R.P. (2006). Speech evaluation with and without palatal obturator in patients submitted to maxillectomy. J. Appl. Oral Sci..

[14] Aramany M.A. (1978). Basic principles of obturator design for partially edentulous patients. Part I: Classification. J. Prosthet. Dent..

[15] Aramany M.A. (1978). Basic principles of obturator design for partially edentulous patients. Part II: Design principles. J. Prosthet. Dent..

[16] Ali M.M., Khalifa N., Alhajj M.N. (2018). Quality of life and problems associated with obturators of patients with maxillectomies. Head Face Med..

[17] Artopoulou I., Karademas E.C., Papadogeorgakis N., Papathanasiou I., Polyzois G. (2017). Effects of sociodemographic, treatment variables, and medical characteristics on quality of life of patients with maxillectomy restored with obturator prostheses. J. Prosthet. Dent..

[18] Ferro K.J., Morgano S.M., Driscoll C.F., Freilich M.A., Guckes A.D., Knoernschild K.L., McGarry T.J., Twain M. (2017). The Glossary of Prosthodontic Terms: Ninth Edition. J. Prosthet. Dent..

[19] Depprich R., Naujoks C., Lind D., Ommerborn M., Meyer U., Kübler N.R., Handschel J. (2011). Evaluation of the quality of life of patients with maxillofacial defects after prosthodontic therapy with obturator prostheses. Int. J. Oral Maxillofac. Surg..

[20] Hanawa S., Kitaoka A., Koyama S., Sasaki K. (2015). Influence of maxillary obturator prostheses on facial morphology in patients with unilateral maxillary defects. J. Prosthet. Dent..

[21] Rieger J.M., Tang J.A.L., Wolfaardt J., Harris J., Seikaly H. (2011). Comparison of speech and aesthetic outcomes in patients with maxillary reconstruction versus maxillary obturators after maxillectomy. J. Otolaryngol. Head Neck Surg..

[22] Irish J., Sandhu N., Simpson C., Wood R., Gilbert R., Gullane P., Brown D., Goldstein D., Devins G., Barker E. (2009). Quality of life in patients with maxillectomy prostheses. Head Neck.

[23] Ortholan C., Benezery K., Dassonville O., Poissonnet G., Bozec A., Guiochet N., Belkacemi Y. (2011). A specific approach for elderly patients with head and neck cancer. Anticancer Drugs.

[24] Moreno M.A., Skoracki R.J., Hanna E.Y., Hanasono M.M. (2010). Microvascular free flap reconstruction versus palatal obturation for maxillectomy defects. Head Neck.

[25] Gupta R., Luthra R., Gupta S. (2016). Evaluation of quality of life of maxillectomy patients after prosthetic obturator rehabilitation. Int. Dent. Med. J. Adv. Res..

[26] Desjardins R.P. (1978). Obturator prosthesis design for acquired maxillary defects. J. Prosthet. Dent..

[27] Keyf F. (2001). Obturator prostheses for hemimaxillectomy patients. J. Oral Rehabil..

[28] Huryn J.M., Piro J.D. (1989). The maxillary immediate surgical obturator prosthesis. J. Prosthet. Dent..

[29] Kar S., Tripathi A. (2016). Treatment outcome with delayed maxillary obturator prosthesis: Case series of four patients. J. Prosthodont..

[30] Tripathi A., Gupta A., Arora V. (2016). Effect of prosthodontic rehabilitation of maxillary defects on hypernasality of speech. J. Prosthodont..

[31] Brandão T.B., Vechiato Filho A.J., de Batista S., de Oliveira M.C.Q., Santos-Silva A.R. (2016). Obturator prostheses versus free tissue transfers: A systematic review of the optimal approach to improving the quality of life for patients with maxillary defects. J. Prosthet. Dent..

[32] Umino S., Masuda G., Ono S., Fujita K. (1998). Speech intelligibility following maxillectomy with and without a prosthesis: An analysis of 54 cases. J. Oral Rehabil..

[33] Parr G.R., Tharp G.E., Rahn A.O. (1989). Prosthodontic principles in the framework design of maxillary obturator prostheses. J. Prosthet. Dent..

[34] Kumar P., Alvi H.A., Rao J., Singh B.P., Jurel S.K., Kumar L., Aggarwal H. (2013). Assessment of the quality of life in maxillectomy patients: A longitudinal study. J. Adv. Prosthodont..

[35] Arigbede A.O., Dosumu O.O., Shaba O.P., Esan T.A. (2006). Evaluation of speech in patients with partial surgically acquired defects: Pre and post prosthetic obturation. J. Contemp. Dent. Pract..

[36] Kornblith A.B., Zlotolow I.M., Gooen J., Huryn J.M., Lerner T., Strong E.W., Shah J.P., Spiro R.H., Holland J.C. (1996). Quality of life of maxillectomy patients using an obturator prosthesis. Head Neck.

[37] Rogers S.N., Lowe D., McNally D., Brown J.S., Vaughan E.D. (2003). Health-related quality of life after maxillectomy: A comparison between prosthetic obturation and free flap. J. Oral Maxillofac. Surg..

[38] Seignemartin C.P., Miranda M.E., Luz J.G.C., Teixeira R.G. (2015). Understandability of speech predicts quality of life among maxillectomy patients restored with obturator prosthesis. J. Oral Maxillofac. Surg..

[39] Hertrampf K., Wenz H.J., Lehmann K.M., Lorenz W., Koller M. (2004). Quality of life of patients with maxillofacial defects after treatment for malignancy. Int. J. Prosthodont..

[40] Kaya M.S., Güçlü B., Schimmel M., Akyüz S. (2017). Two-colour chewing gum mixing ability test for evaluating masticatory performance in children with mixed dentition: Validity and reliability study. J. Oral Rehabil..

[41] Schimmel M., Christou P., Miyazaki H., Halazonetis D., Herrmann F.R., Müller F. (2015). A novel colourimetric technique to assess chewing function using two-coloured specimens: Validation and application. J. Dent..

[42] van der Bilt A., Mojet J., Tekamp F.A., Abbink J.H. (2010). Comparing masticatory performance and mixing ability. J. Oral. Rehabil..

[43] Antoniou D.V., Toljanic J.A., Graham L. (1996). Obturator prosthesis retention for edentulous patients with large palatal defects: A clinical report. J. Prosthet. Dent..

[44] Favia G., Corsalini M., Di Venere D., Pettini F., Favia G., Capodiferro S., Maiorano E. (2013). Immunohistochemical evaluation of neuroreceptors in healthy and pathological temporo-mandibular joint. Int. J. Med. Sci..

[45] Koyama S., Sasaki K., Inai T., Watanabe M. (2005). Effects of defect configuration, size, and remaining teeth on masticatory function in post-maxillectomy patients. J. Oral Rehabil..

[46] Kapur K.K. (1967). A clinical evaluation of denture adhesives. J. Prosthet. Dent..

[47] Corsalini M., Di Venere D., Sportelli P., Magazzino D., Ripa C., Cantatore F., Cagnetta G., de Rinaldis C., Montemurro N., de Giacomo A. (2018). Evaluation of prosthetic quality and masticatory efficiency in patients with total removable prosthesis study of 12 cases. Oral Implantol..

[48] Corsalini M., Di Venere D., Stefanachi G., Muci G., Palminteri A., Laforgia A., Pettini F. (2017). Maxillary overdenture retained with an implant support CAD-CAM bar: A 4 years follow up case. Open Dent. J..

[49] Kreeft A.M., Krap M., Wismeijer D., Speksnijder C.M., Smeele L.E., Bosch S.D., Muijen M.S., Balm A.J. (2012). Oral function after maxillectomy and reconstruction with an obturator. Int. J. Oral Maxillofac. Surg..

